# Interaction of Heavy Metals (Cadmium and Selenium) in an Experimental Study on Goldfish: Hematobiochemical Changes and Oxidative Stress

**DOI:** 10.3390/jox15020057

**Published:** 2025-04-16

**Authors:** Yasaman Aghaei Hashtjin, Mahdieh Raeeszadeh, Ali Parsa Khanghah

**Affiliations:** 1 Graduate of Faculty of Veterinary Sciences, Sa.C., Islamic Azad University, Sanandaj 618, Iran; yasagaei.24@gmail.com; 2 Department of Basic Sciences, Sa.C., Islamic Azad University, Sanandaj 618, Iran; 3 Department of Aquatic Animal Health and Disease, Sa.C., Islamic Azad University, Sanandaj 618, Iran; a.parsa@ausdj.ac.ir

**Keywords:** physiological performance, selenium, cadmium, oxidative stress, goldfish

## Abstract

Background: Heavy metal interactions within aquatic ecosystems significantly affect fish physiology. This study evaluated the protective role of selenium against cadmium-induced hematological, biochemical, and electrophoretic alterations in goldfish. Methods: A total of 120 goldfish individuals were divided into four groups: control, cadmium chloride-treated (2.8 mg/L), sodium selenite-treated (2 mg/L), and a combined cadmium and selenium-treated group. After 14 days, blood samples were collected and analyzed for hematological parameters, biochemical markers, and serum protein electrophoresis. Results: Cadmium exposure led to significant reductions in red blood cell (RBC) and white blood cell (WBC) counts, hemoglobin (Hb), and hematocrit (HCT) (*p* < 0.001). Selenium supplementation alleviated these declines and improved overall hematological function. Additionally, cadmium exposure decreased albumin and total protein levels while elevating aspartate aminotransferase (AST) and alanine aminotransferase (ALT) levels, indicating liver damage. Selenium co-treatment reduced cadmium accumulation and mitigated liver toxicity. Elevated urea and creatinine levels in cadmium-exposed fish were also significantly lowered in the combined treatment group (*p* < 0.0001). Furthermore, selenium supplementation enhanced antioxidant defense mechanisms by increasing catalase (CAT), superoxide dismutase (SOD), and glutathione peroxidase (GPx) activity while reducing malondialdehyde (MDA) levels, effectively counteracting cadmium-induced oxidative stress. Conclusion: Sodium selenite at a dose of 2 mg/L effectively mitigated the toxic effects of cadmium chloride on hematological, biochemical, and oxidative stress markers in goldfish, demonstrating its protective potential against heavy metal toxicity.

## 1. Introduction

Exposure to xenobiotics, particularly heavy metals, presents a significant health challenge for living organisms [1]. These metals can interact with biological systems either individually or in combination, accumulating primarily in vital organs, such as the liver, kidneys, brain, and hematopoietic tissues. This accumulation triggers molecular toxicity mechanisms, posing serious health risks not only to the affected organisms but also to human populations that consume them [2].

Goldfish (*Carassius auratus*) is widely used as a model species in experimental studies on heavy metal toxicity. Additionally, it is well recognized as an ornamental fish species [3].

Cadmium (Cd) is a major environmental pollutant with diverse toxic effects [4]. These effects result from multiple interconnected toxicity mechanisms, making it challenging to delineate them precisely [5]. The complexity of Cd toxicity arises from the overlapping nature of these pathways. The primary mechanisms through which Cd exerts its harmful effects include alterations in gene expression, disruption of DNA repair processes, interference with apoptosis and autophagy, induction of oxidative stress, and interactions with essential elements [6].

Numerous molecular mechanisms have been identified in Cd toxicity, including DNA damage, cellular apoptosis, oxidative stress induction, depletion of enzymatic antioxidants, and disruptions in energy metabolism and growth pathways [7]. These chronic effects can significantly impact ecosystems, leading to long-term ecological consequences [8].

Another key aspect of Cd toxicity is its interference with essential heavy metals such as calcium, selenium, and zinc. This interaction alters their absorption and biological efficacy, further exacerbating Cd-induced toxicity [9].

Selenium, a heavy metal, naturally occurs in varying concentrations across ecosystems. While trace amounts of selenium may be present in surface waters, it primarily accumulates in sediments, where it enters the bodies of aquatic organisms, particularly benthic species, such as cyprinids. In aquatic toxicology, selenium presents a unique paradox, functioning both as an essential nutrient and a potential toxin [10]. It plays a crucial role in the synthesis and proper functioning of glutathione peroxidase, a key cellular antioxidant enzyme that protects cell membranes from oxidative damage caused by lipid peroxidation [11].

Selenium deficiency can impair cellular metabolism and function due to peroxide accumulation, a byproduct of digestion. In fish, selenium deficiency manifests as reduced growth, anemia, muscular dystrophy, and increased mortality [12]. Conversely, selenium has been reported to act as an antioxidant, mitigating oxidative stress and counteracting the toxic effects of heavy metals. The beneficial effects of adequate selenium levels in fish diets are well-documented [13].

Selenium absorption in fish occurs via two primary pathways: direct uptake from water through the gills and dietary intake through the digestive tract [14]. However, excessive selenium accumulation in tissues can lead to toxicity, resulting in tissue and gill necrosis, impaired growth, reproductive dysfunction, and ultimately increased mortality [10].

Additionally, selenium has been reported to exhibit antagonistic, plasma protein-binding interaction, and chelating effects against toxic heavy metals such as arsenic and cadmium. These interactions suggest a potential protective role of selenium in mitigating heavy metal toxicity and reducing cellular damage in living organisms [15].

Given the importance of understanding the complex interactions of heavy metals in living organisms, particularly aquatic species, this study aimed to assess the subacute toxicity induced by selenium and cadmium. In this study, we examined the biochemical and hematological changes, the oxidative stress enzyme activity, and the serum protein electrophoresis profiles under combined exposure to selenium and cadmium. The results provide valuable insights into the synergistic and antagonistic interactions between essential and toxic heavy metals, enhancing our understanding of their effects in aquatic environments.

## 2. Materials and Methods

### 2.1. Experimental Design

Initially, 120 live goldfish (*Carassius auratus*) individuals with an average weight of 20 ± 8 g were purchased in Spring 2024 from a fish farm in Gilan Province and transported to the laboratory of the Veterinary Faculty in Sanandaj. After a 10-day acclimatization period, the fish were randomly divided into four groups of 10 fish individuals, with three replicates. They were housed in four aerated aquariums, each filled with 30 L of well water at 20 ± 2 °C and a pH range of 7.5–8.5. All procedures followed standard international guidelines for animal care and were approved by the Ethics Committee of Islamic Azad University, Sanandaj, under approval code IR.IAU.SDJ.REC.1402.143.

The groups were treated as follows:

1. Control Group: No treatment.
2. *cad* Group (Cadmium exposure): Exposed to cadmium chloride (Sigma-Aldrich, St. Louis, MO, USA Company) at 2.8 mg/L (LD50 cadmium 11.2 mg/L in goldfish individuals) [14].
3. *sel* Group (Selenium exposure): Exposed to sodium selenite (Sigma-Aldrich, USA) at 2 mg/L [15].
4. *cad*+*sel* Group: Exposed simultaneously to cadmium chloride (2.8 mg/L) and sodium selenite (2 mg/L).

The water in the aquariums was replaced every three days. Replacement water was stored in a tank for several hours before use to ensure thermal equilibration with the environment. Fish were fed twice daily with flake food (Behparvar, Tehran, Iran) at 3% of their body weight. Water in the different groups was filtered by siphoning.

After 14 days of exposure, blood samples were collected under anesthesia using 2 mL heparinized syringes at a 45° angle from the caudal vein. Approximately 1.5 mL of blood was collected in EDTA-containing tubes for hematological analysis, and the rest was placed in clotting tubes for biochemical and serum protein electrophoresis analysis. The samples were immediately transported to the laboratory for analysis.

### 2.2. Hematological Analysis

The red blood cell (RBC) and white blood cell (WBC) counts were determined using a Neubauer chamber and red/white diluting fluids [16]. Hematocrit (Hct) was measured by the microhematocrit method [17]. Hemoglobin (Hb) levels were assessed using the cyanmethemoglobin method with a commercial kit (Zist Azma, Tehran, Iran) [18].

### 2.3. Biochemical Analysis

Blood samples in tubes without anticoagulant were centrifuged for 15 min at 6000 rpm to separate serum, which was stored in microtubes for analysis. Serum levels of alanine aminotransferase (ALT), aspartate aminotransferase (AST), glucose, cholesterol, urea, and creatinine were measured using an Autoanalyzer (Eppendorf, EPOS, Hamburg, Germany) [19,20]. Levels of malondialdehyde (MDA), glutathione peroxidase (GPx), superoxide dismutase (SOD), and catalase (CAT) were measured using specific kits (Novand Salamat, Urmia, Iran) via refractometric methods [21].

### 2.4. Serum Protein Electrophoresis

The levels of serum proteins, including albumin, globulin, alpha-1, alpha-2, beta, gamma, and total protein, were analyzed using vertical electrophoresis [22].

### 2.5. Statistical Analysis

The results of the data analysis are presented. Given the quantitative nature of the examined variables, descriptive statistics, including mean and standard deviation, were utilized for data summarization. A one-way analysis of variance was performed to compare the variables among the study groups. In cases where ANOVA indicated statistical significance, Tukey’s post hoc test was conducted to identify significant intergroup differences. The normality of the data was assessed using the Kolmogorov–Smirnov test, along with skewness and kurtosis indices. Once the necessary assumptions were confirmed, ANOVA was applied for further analysis. Data were analyzed using SPSS version 22, with a significance level set at 0.05. For better visualization, graphical representations were included, and all figures were generated using GraphPad Prism 10.

## 3. Results

**Figure 1:**
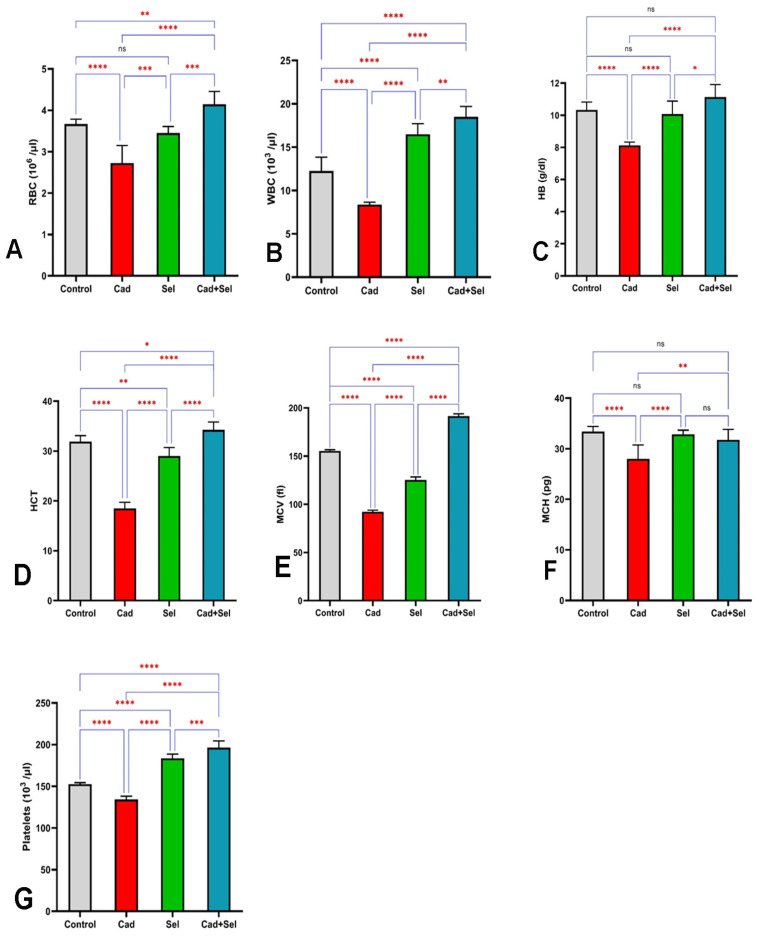
Changes in hematological parameters (RBC, WBC, Hb, HCT, MCV, MCH, and platelet, respectively) in the blood of different experimental groups in figures (**A**–**G**). The differences between groups are indicated by the asterisks. Statistical significance levels are determined based on the stars. ns: not significant, *: *p* < 0.05, **: *p* < 0.01, ***: *p* < 0.001, ****: *p* < 0.0001.

Figure 1A–C illustrates the variations in RBC, WBC, and Hb levels among the study groups. The analysis of RBC levels revealed a significant difference between the groups (*p* < 0.001), with the lowest mean RBC count observed in the *cad* group (2.73 ± 0.43) × 10^6^/µL, while the highest was recorded in the *cad*+*sel* group (4.15 ± 0.31) × 10^6^/µL. No significant difference was found between the control and *sel* groups.

Similarly, WBC levels varied significantly across the groups (*p* < 0.001). Pairwise comparisons showed that the mean WBC count differed significantly among all groups, with the lowest value in the *cad* group and the highest in the *cad*+*sel* group.

Regarding Hb levels, the *cad* group exhibited a significantly lower mean Hb concentration (8.13 ± 0.21 g/dL) compared to the other groups, while the highest mean Hb was observed in the *cad*+*sel* group (11.13 ± 0.79 g/dL), with a statistically significant difference (*p* < 0.001).

Hematocrit (HCT) levels exhibited significant variations among the study groups (*p* < 0.001). The lowest mean HCT was recorded in the *cad* group (18.5 ± 1.20), which was significantly lower than in all other groups. Conversely, the highest HCT level was observed in the *cad*+*sel* group, showing a statistically significant increase compared to the other groups (Figure 1D).

Similarly, mean MCV varied significantly among the groups (*p* < 0.001). The *cad* group demonstrated the lowest MCV (92.25 ± 1.58 fl), while the highest value was recorded in the *cad*+*sel* group (191.4 ± 2.45 fl), followed by the *sel* group (125.25 ± 3.15 fl).

Mean corpuscular hemoglobin (MCH) was lowest in the *cad* group (28.00 ± 2.73 pg). However, the differences in MCH among the control, *sel*, and *cad*+*sel* groups were not statistically significant.

Regarding platelet (PLT) levels, the lowest count was observed in the *cad* group, while the highest values were recorded in the *sel* and *cad*+*sel* groups, with statistically significant differences between them (Figure 1E–G).

**Figure 2:**
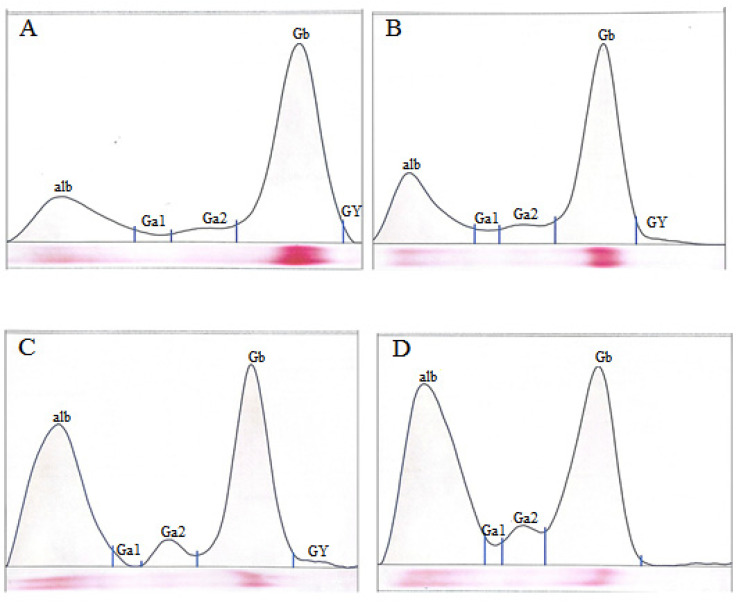
Electrophoretic pattern of serum proteins (albumin and globulins) in the four experimental groups. alb (albumin), G (globulin).

The electrophoretic analysis of serum proteins revealed significant variations among the study groups. The highest albumin concentration was recorded in the cadmium combined with selenium *cad*+*sel* group (1.90 ± 0.07 g/dL), followed by the *sel* group (1.85 ± 0.12 g/dL). In contrast, the lowest albumin concentration was observed in the *cad* group, with a mean value of 0.70 ± 0.17 g/dL. Conversely, the highest globulin concentration was found in the control group (2.82 ± 0.21 g/dL), whereas the lowest levels were detected in the *sel* group (2.00 ± 0.08 g/dL), followed closely by the *cad*+*sel* group (1.97 ± 0.09 g/dL). The albumin-to-globulin (A/G) ratio was significantly lower in the *cad* group compared to all other groups. However, the highest A/G ratio was recorded in the sel group, followed by the *cad*+*sel* group, with statistically significant differences among the groups (*p* < 0.001). Furthermore, the levels of alpha-1, alpha-2, beta, and gamma globulins were lowest in the *cad* group, whereas the highest concentrations were observed in the *cad*+*sel* group (Figure 2A–D).

**Figure 3:**
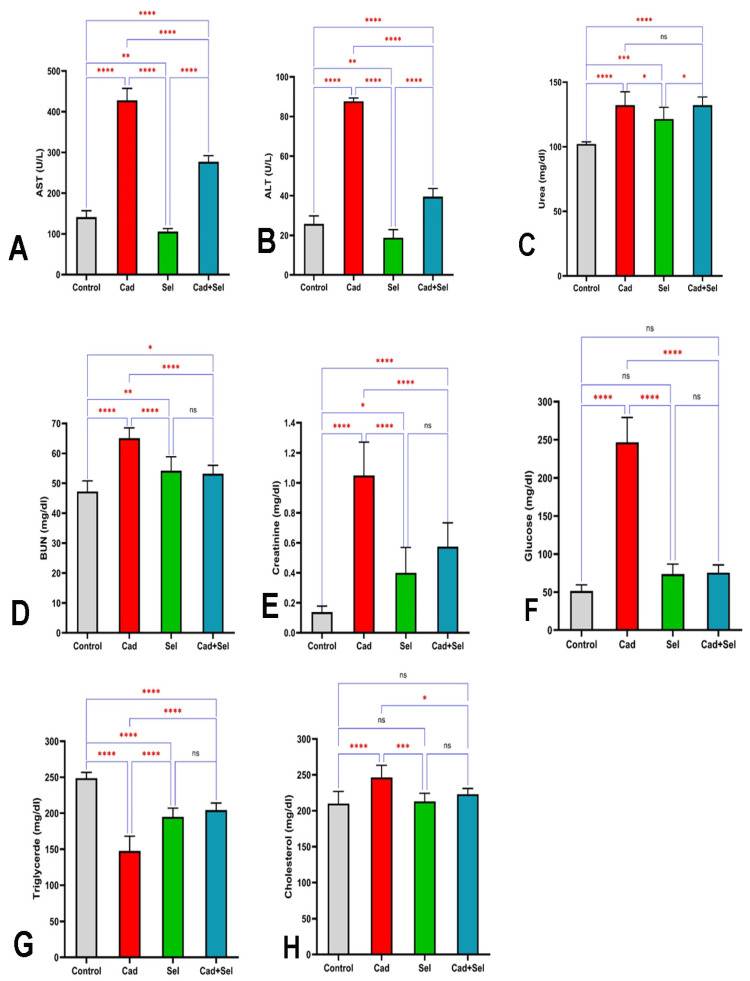
Changes in liver (**A**,**B**) and kidney (**C**,**D**) biochemical parameters and serum metabolic indices (**E**–**H**) in the experimental groups. The differences between groups are indicated by the asterisks. Statistical significance levels are determined based on the stars. ns: not significant, *: *p* < 0.05, **: *p* < 0.01, ***: *p* < 0.001, ****: *p* < 0.0001.

Figure 3A,B illustrate that the highest liver damage, along with elevated liver enzymes AST and ALT, was observed in the *cad* group, while the lowest values were recorded in the *sel* group. These differences were statistically significant at *p* < 0.001 level compared to other groups.

Figure 3C,D display the levels of urea and BUN. The highest mean urea (132.25 ± 10.32 mg/dL) and BUN (65.00 ± 3.46 mg/dL) were observed in the *cad* group, while the lowest urea and BUN levels were recorded in the control group, with values of 102.25 ± 1.25 mg/dL and 47.25 ± 3.58 mg/dL, respectively.

Kidney function, as indicated by creatinine levels (Figure 3E), showed that creatinine in the *cad* group was significantly higher than in other groups, while the control group exhibited significantly lower levels (*p* < 0.0001). Meanwhile, the mean creatinine levels in the *sel* and *cad*+*sel* groups were not significantly different from each other.

For serum glucose levels, the highest value was recorded in the *cad* group (246.75 ± 32.46 mg/dL), and the lowest was found in the control group (51.50 ± 8.05 mg/dL). The mean glucose levels in other groups showed no significant differences (Figure 3F).

In contrast, triglyceride levels were lowest in the *cad* group (147.50 ± 20.53 mg/dL) and highest in the control group (248.75 ± 7.91 mg/dL). The intergroup differences were statistically significant (*p* < 0.001) (Figure 3G).

The serum cholesterol levels of different groups are depicted in Figure 3H. The mean cholesterol levels varied significantly among the groups (*p* < 0.001). The *cad* group had significantly higher cholesterol levels compared to other groups, while the lowest levels were observed in the *sel* and control groups (*p* < 0.0001). There were no statistically significant differences in cholesterol levels among the other groups (Figure 3).

**Figure 4:**
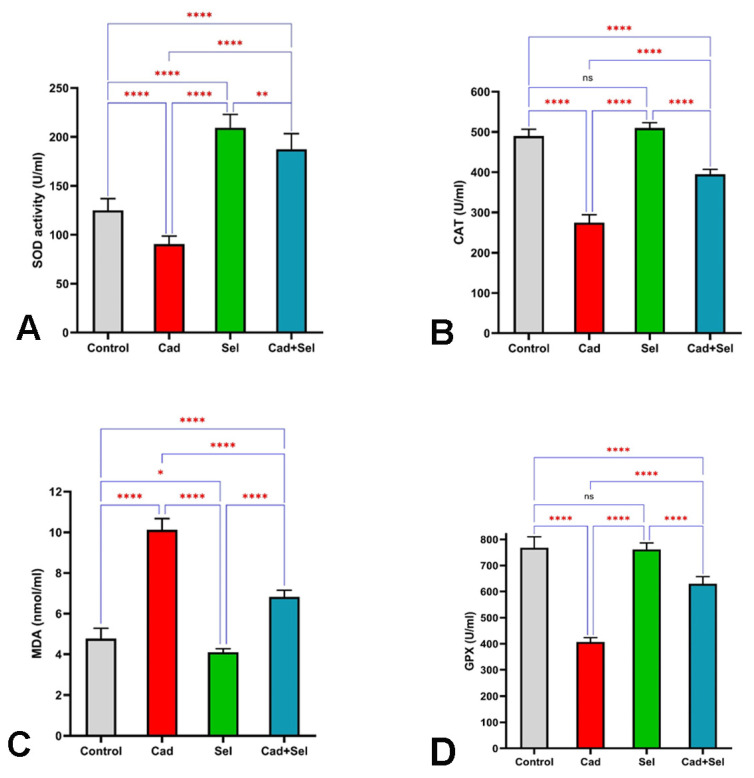
Changes in antioxidant enzyme activities (SOD, CAT, and GPx) and MDA levels in the experimental groups in the (**A**–**D**) figures. ns: not significant, *: *p* < 0.05, **: *p* < 0.01, ****: *p* < 0.0001.

Figure 4A–D present the oxidative stress parameters in serum, highlighting significant variations among the study groups.

Superoxide dismutase (SOD) activity differed significantly among the groups (*p* < 0.001). The highest SOD activity was recorded in the *sel* group, with a mean of 209.50 ± 13.55 U/mL, whereas the lowest activity was observed in the *cad* group (90.50 ± 8.16 U/mL). The cadmium combined with the *cad*+*sel* group exhibited an intermediate SOD activity of 187.50 ± 15.81 U/mL, with statistically significant differences among the groups (Figure 4A).

Figure 4B illustrates the activity of CAT, an essential antioxidant enzyme. The highest CAT activity was observed in the *sel* group (510 ± 13.09 U/mL), while the lowest activity was recorded in the *cad* group (275.00 ± 19.27 U/mL), indicating a significant reduction due to cadmium exposure.

Glutathione peroxidase (GPx) activity is presented in Figure 4C. The *cad* group exhibited significantly lower GPx activity compared to the other groups. No significant differences were detected between the control and *sel* groups. However, the GPx activity in the *cad*+*sel* group differed significantly from that in all other groups (*p* < 0.0001).

Figure 4D illustrates the MDA levels across the study groups. The mean MDA concentration was significantly higher in the *cad* group (10.13 ± 0.55 nmol/mL) compared to the *sel* group (4.10 ± 0.17 nmol/mL), indicating the oxidative damage induced by cadmium exposure. These differences were statistically significant when compared to the other experimental groups (*p* < 0.001).

## 4. Discussion

In this study, the effect of Se on Cd toxicity, which has been previously addressed to some extent in past studies, was experimentally evaluated in aquatic organisms. This study focused on hematological parameters, hepatic and renal biochemical changes, and alterations in blood proteins, including albumin and globulin. Ultimately, these changes were correlated with oxidative damage and antioxidant enzyme activities.

One of the key findings of this study was the reduction in RBCs, Hb, and HCT due to the disruption of erythropoiesis in the spleen and renal erythropoietin production under Cd exposure. However, supplementation with selenium led to an increase in these parameters, suggesting an antagonistic role of selenium against cadmium toxicity. The observed decline in WBCs under Cd exposure emphasized the immunosuppressive effects of this heavy metal [16,17], whereas selenium exhibited immunostimulatory effects, compensating for the Cd-induced immunosuppression [18].

Cadmium-induced stress has been reported to cause macrocytic anemia by either damaging mature RBCs or inhibiting erythropoiesis and angiogenesis, ultimately leading to anemia in fish [19]. Fish subjected to stress conditions typically show a reduction in RBC count, HCT levels, and Hb concentration. This stress-induced anemia serves as a key indicator for assessing environmental stress [20].

Following absorption, Cd predominantly accumulates in the liver, kidneys, bones, and subsequently in the lungs. While the cellular effects of Cd have been extensively documented, the underlying molecular mechanisms remain unclear [21]. The liver plays a crucial role in Cd detoxification through diverse mechanisms [22].

Another significant finding of this study was the decrease in serum protein levels, including albumin, along with an increase in globulin levels, resulting in a reduced albumin-to-globulin ratio in the Cd-exposed group. However, selenium administration led to an increase in these parameters. Additionally, liver enzymes AST and ALT reached their highest serum concentrations in the Cd group. The accumulation of Cd in the liver, kidneys, and spleen, as previously reported in the literature, contributes to liver damage, increased enzyme levels, and consequently reduced albumin synthesis, leading to a decreased albumin-to-globulin ratio [17,23].

The elevated levels of urea, BUN, and creatinine in the Cd group, reaching peak serum concentrations, and their subsequent significant reduction upon co-exposure to selenium highlight the contrasting effects of this essential metal. Selenium exhibited protective effects by modulating Cd metabolism, reducing its accumulation in tissues, and improving physiological functions of the liver and kidneys [6].

Selenium protects cells from Cd-induced toxicity by reducing ROS levels and enhancing the activity of antioxidant selenoproteins such as GPx [24]. The primary protective mechanism of selenium against arsenic (As) and Cd in animals is the sequestration of these toxic elements into biologically inert complexes. Well-known antioxidant selenoenzymes, including GPx and thioredoxin reductase (TrxR), also contribute to selenium-dependent detoxification of As and Cd, although they likely play a secondary role [15].

The functionality of selenium in animals largely depends on the expression of selenoproteins. This small yet essential group of proteins includes GPxs and TrxRs, which regulate redox homeostasis and antioxidant defense, as well as iodothyronine deiodinases (DIOs), which are essential for thyroid hormone synthesis and metabolism [25]. Notably, the antioxidant activity that eliminates ROS and mitigates oxidative stress-induced cellular damage is not only exhibited by GPx and TrxR but also by other selenoproteins, such as selenoproteins P, R, and W [26]. The synthesis of selenoproteins is primarily regulated by dietary selenium intake, but in selenium-deficient conditions, there is a tissue-specific hierarchy in selenoprotein expression [27].

Plasma proteins have been shown to bind to various heavy metals, thereby influencing their transport, distribution, elimination, and toxicological effects on the human body [12]. Certain plasma proteins, such as metallothioneins and selenoproteins, play critical roles in mitigating heavy metal toxicity by binding these metals and preventing their interaction with target tissues [28]. Carrier proteins such as albumin and transferrin, for example, have specific functions in the transport of metal compounds in the bloodstream [29].

Another novel finding of this study was the significant reduction in antioxidant enzyme activity in the Cd-exposed group, whereas increased activity was observed in the selenium and Cd + Se groups. Previous studies have demonstrated that selenium treatment enhances GSH-Px, SOD, and CAT levels while mitigating Cd-induced oxidative stress.

A study conducted by Choi et al. (2015) on goldfish populations revealed that toxic stress markers, such as metallothionein (MT) and aspartate aminotransferase, as well as other stress-related parameters, including corticotropin-releasing hormone (CRH), plasma ACTH, cortisol, glucose, hydrogen peroxide (H_2_O_2_), and lipid peroxidation (LPO), were significantly elevated in fish exposed to 3–4 mg/L selenium compared to those exposed to lower concentrations (0–2 mg/L) [30]. These findings indicated that selenium concentrations of 3–4 mg/L could induce acute physiological and toxic stress responses under aquarium conditions.

The present study demonstrates that sublethal concentrations of Cd significantly affect plasma protein and glucose levels [31]. With increasing Cd concentrations in the environment, plasma protein levels decreased, while glucose levels increased. The observed hyperglycemia in fish may be attributed to excessive mucus secretion, tissue swelling, and necrosis at the gill surface, which occurs in response to heavy metal pollution, such as Cd exposure [32,33].

Previous studies have reported that a significant reduction in triglyceride levels may result from damage to intestinal villi or hepatic tissue [34]. Heavy metals such as Cd generate substantial amounts of ROS, leading to oxidative stress and increased lipid peroxidation products [35]. It has been established that Cd negatively impacts lipid profiles and lipoproteins, causing tissue damage through lipid peroxidation [36].

## 5. Conclusions

Recent studies have demonstrated that exposure to cadmium under experimental conditions simulating an aquatic ecosystem can cause severe damage to hematopoietic tissues, including the spleen, as well as critical organs such as the liver and kidneys. The detrimental effects of cadmium toxicity manifest as anemia, affecting RBC count, Hb levels, and HCT, along with immunosuppression characterized by a reduction in WBC count and elevated levels of hepatic enzymes, urea, and creatinine. Notably, selenium supplementation has been identified as a potential protective agent, mitigating these toxic effects through its antagonistic properties.

Cadmium poisoning is also associated with a decline in serum albumin levels, which leads to an increase in the bioavailable free form of cadmium, exacerbating tissue damage in organs where it accumulates. Selenium, through its antioxidant properties at optimal doses, can counteract cadmium-induced oxidative stress by enhancing GPx activity and reducing malondialdehyde levels, thereby mitigating cadmium-induced molecular toxicity.

Further exploration of the molecular and inflammatory pathways, as well as the expression of genes involved in cadmium toxicity, is crucial to comprehensively elucidate the underlying mechanisms. Additionally, optimizing dietary selenium levels in aquatic organisms may serve as an effective strategy to attenuate the toxic effects of heavy metals such as cadmium and arsenic through antagonistic and chelating mechanisms. This approach could significantly contribute to improving the health outcomes in both aquatic animals and humans by reducing the impact of xenobiotic exposure.

## Data Availability

The datasets used and/or analyzed during the current study are available from the corresponding author upon reasonable request.

## Abbreviations

MT	Metallothionein
CRH	Corticotropin-releasing hormone
LPO	Lipid peroxidation
Ht	Hematocrit

## References

[1] Wu X., Cobbina S.J., Mao G., Xu H., Zhang Z., Yang L. (2016). A review of toxicity and mechanisms of individual and mixtures of heavy metals in the environment. Environ. Sci. Pollut. Res..

[2] Ali H., Khan E., Ilahi I. (2019). Environmental chemistry and ecotoxicology of hazardous heavy metals: Environmental persistence, toxicity, and bioaccumulation. J. Chem..

[3] Ali Z., Sher N., Muhammad I., Nayab G.E., Alouffi A., Almutairi M.M., Khan I., Ali A. (2025). The combined effect of cadmium and copper induces bioaccumulation, and toxicity and disrupts the antioxidant enzymatic activities of goldfish (*Carassius auratus*). Toxicol. Rep..

[4] Genchi G., Sinicropi M.S., Lauria G., Carocci A., Catalano A. (2020). The effects of cadmium toxicity. Int. J. Environ. Res. Public Health.

[5] Wang M., Chen Z., Song W., Hong D., Huang L., Li Y. (2021). A review on cadmium exposure in the population and intervention strategies against cadmium toxicity. Bull. Environ. Contam. Toxicol..

[6] Teschke R. (2024). Copper, iron, cadmium, and arsenic, all generated in the universe: Elucidating their environmental impact risk on human health including clinical liver injury. Int. J. Mol. Sci..

[7] Unsal V., Dalkıran T., Çiçek M., Kölükçü E. (2020). The role of natural antioxidants against reactive oxygen species produced by cadmium toxicity: A review. Adv. Pharm. Bull..

[8] Souza-Arroyo V., Fabián J.J., Bucio-Ortiz L., Miranda-Labra R.U., Gomez-Quiroz L.E., Gutiérrez-Ruiz M.C. (2022). The mechanism of the cadmium-induced toxicity and cellular response in the liver. Toxicology.

[9] Parui R., Nongthombam G.S., Hossain M., Adil L.R., Gogoi R., Bhowmik S., Barman D., Iyer P.K. (2024). Impact of heavy metals on human health. Remediation of Heavy Metals: Sustainable Technologies and Recent Advances.

[10] Uddin M.H., Ritu J.R., Putnala S.K., Rachamalla M., Chivers D.P., Niyogi S. (2024). Selenium toxicity in fishes: A current perspective. Chemosphere.

[11] Li Z.-M., Wang X.-L., Jin X.-M., Huang J.-Q., Wang L.-S. (2023). The effect of selenium on antioxidant system in aquaculture animals. Front. Physiol..

[12] Minich W.B. (2022). Selenium metabolism and biosynthesis of selenoproteins in the human body. Biochemistry.

[13] Antia M., Ezejiofor A.N., Orish C.N., Ugwu T., Cirovic A., Cirovic A., Ajibo D.N., Orisakwe O.E. (2024). Zn and Se supplementation abrogated metals-(metaloids) mixture mediated ocular-thymus toxicity via modulation of oxido-inflammatory and antiapoptotic mechanisms in female Sprague Dawley rats. Eur. J. Clin. Exp. Med..

[14] Sumana S.L., Chen H., Shui Y., Zhang C., Yu F., Zhu J., Su S. (2023). Effect of dietary selenium on the growth and immune systems of fish. Animals.

[15] Zwolak I. (2020). The role of selenium in arsenic and cadmium toxicity: An updated review of scientific literature. Biol. Trace Elem. Res..

[16] Chen J., Xu Y., Han Q., Yao Y., Xing H., Teng X. (2019). Immunosuppression, oxidative stress, and glycometabolism disorder caused by cadmium in common carp (*Cyprinus carpio* L.): Application of transcriptome analysis in risk assessment of environmental contaminant cadmium. J. Hazard. Mater..

[17] Borgia V., Thatheyus A., Murugesan A. (2019). Impact of electroplating industry effluent on the electrophoretic protein pattern of serum in the freshwater fish Cyprinus carpio. Indian. J. Biochem. Biophys..

[18] Razaghi A., Poorebrahim M., Sarhan D., Björnstedt M. (2021). Selenium stimulates the antitumour immunity: Insights to future research. Eur. J. Cancer.

[19] Yu Y.-B., Lee J.-W., Jo A.-H., Choi Y.J., Choi C.Y., Kang J.-C., Kim J.-H. (2024). Toxic Effects of Cadmium Exposure on Hematological and Plasma Biochemical Parameters in Fish: A Review. Toxics.

[20] Shahjahan M., Islam M.J., Hossain M.T., Mishu M.A., Hasan J., Brown C. (2022). Blood biomarkers as diagnostic tools: An overview of climate-driven stress responses in fish. Sci. Total Environ..

[21] Gong P., Chen F., Liu X., Gong X., Wang J., Ma Y. (2012). Protective effect of caffeic acid phenethyl ester against cadmium-induced renal damage in mice. J. Toxicol. Sci..

[22] Arroyo V., Flores K., Ortiz L., Gómez-Quiroz L., Gutiérrez-Ruiz M. (2012). Liver and cadmium toxicity. J. Drug Metab. Toxicol. S.

[23] de la Iglesia F.A., Haskins J.R., Feuer G. (2003). Hepatotoxicity of cardiovascular and antidiabetic drugs. Drug-Induced Liver Disease.

[24] Zhang C., Ge J., Lv M., Zhang Q., Talukder M., Li J.-L. (2020). Selenium prevent cadmium-induced hepatotoxicity through modulation of endoplasmic reticulum-resident selenoproteins and attenuation of endoplasmic reticulum stress. Environ. Pollut..

[25] Gribling A., Wurth L., Verheggen C., Leichter M., Krol A., Bertrand E., Allmang C. (2013). Selenoprotein mRNA cap hypermethylation and translation initiation. Микрoэлементы в медицине.

[26] Chaudière J. (2023). Biological and catalytic properties of selenoproteins. Int. J. Mol. Sci..

[27] Bulteau A.-L., Chavatte L. (2015). Update on selenoprotein biosynthesis. Antioxid. Redox Signal..

[28] Sharma P., Srivastava V., Kumar A., Misra A.N. (2018). Mechanisms of metalloid uptake, transport, toxicity, and tolerance in plants. Emerging Trends of Plant Physiology for Sustainable Crop Production Apple.

[29] Ajayi N.D., Ajayi S.A., Oladoyinbo O.B., Olaniyi O.O. (2024). A review of literature on transferrin: Deciphering its complex mechanism in cellular iron regulation and clinical implications. Asian J. Res. Infect. Dis..

[30] Choi Y.J., Yang S.-G., Jung M.-M., Kim B.-S., Yun S.G., Choi C.Y. (2015). Effects of waterborne selenium on toxic and physiological stress response in goldfish, Carassius auratus. Mol. Cell. Toxicol..

[31] Pi J., Li X., Zhang T., Li D. (2016). Effects of acute exposure to sublethal waterborne cadmium on energy homeostasis in silver carp (*Hypophthalmichthys molitrix*). Bull. Environ. Contam. Toxicol..

[32] Shahjahan M., Taslima K., Rahman M.S., Al-Emran M., Alam S.I., Faggio C. (2022). Effects of heavy metals on fish physiology–a review. Chemosphere.

[33] Thamizhazhagan V., Baranitharan M., Sridhar N., Senthilmurugan S. (2020). Hematopathology and histolopathology alteration exposure periods of C4H10NO3PS in freshwater Channa pinctata. Asian J. Adv. Res..

[34] Li X., Liu Q., Pan Y., Chen S., Zhao Y., Hu Y. (2023). New insights into the role of dietary triglyceride absorption in obesity and metabolic diseases. Front. Pharmacol..

[35] Mansoor S., Ali A., Kour N., Bornhorst J., AlHarbi K., Rinklebe J., Abd El Moneim D., Ahmad P., Chung Y.S. (2023). Heavy metal induced oxidative stress mitigation and ROS scavenging in plants. Plants.

[36] El-Demerdash F.M., Nasr H.M. (2014). Antioxidant effect of selenium on lipid peroxidation, hyperlipidemia and biochemical parameters in rats exposed to diazinon. J. Trace Elem. Med. Biol..

